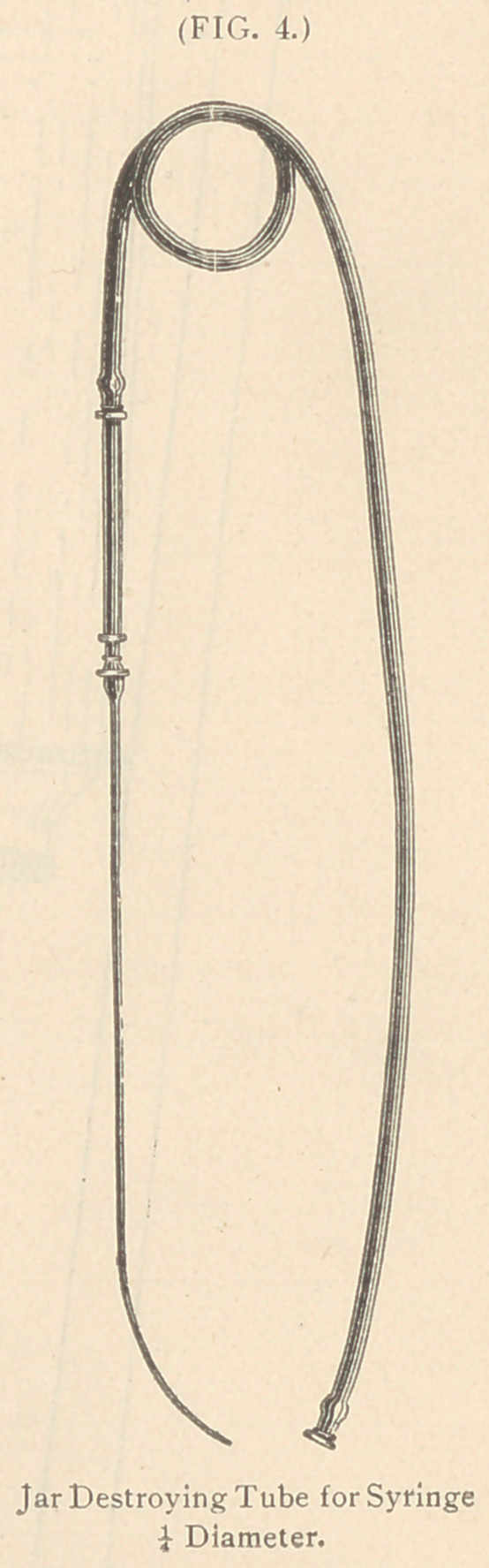# Current News and Opinion

**Published:** 1885-09

**Authors:** 


COPPER AND LEAD IN FOOD.

A. Gautier shows that copper is little calculated to produce mortal results. The solubility of most of its salts, their marked color, nauseating taste, and emetic action give at once warning. The salts of lead, on the contrary, have no pronounced taste, or are even sweetish. They are in general colorless. If introduced into the system, there is no alarming effect until the nervous centers, the liver, and the blood have become interpenetrated with the poison.

### Current News and Opinion.

### THE ASSIMILATION OF IRON.

The following is so apropos to the discussion in the American Dental Association, reported in this number, that we give it place.—Editor.

Later investigations have shown conclusively that the iron administered by physicians to chlorotic patients for the purpose of forming hæmoglobin is not, even in part, absorbed. Experiments upon animals prove that, should iron salts enter the blood, toxic symptoms, similar to arsenical, would ensue. There confronts us then, this question : In what form is iron absorbed and assimilated under normal conditions ? How is hæmoglobin formed ? Milk, the exclusive food for the suckling, must contain the materials for the formation of hæmoglobin, as must also the vitellus, from whose constituents hæmoglobin is formed in the early-development period, in each case without any extraneous sources of formation. In his investigation of milk, the author encountered numerous difficulties. With hen's eggs, however, he succeeded in isolating the iron combinations in question. Upon extracting the vitellus with ether and alcohol, all the iron remained in the undissolved residue. In this residue the iron was found only in *organic* combinations. The conclusion is therefore reached that our food contains no inorganic compounds, but that it exists only in complex organic compounds *which have been formed by the vital processes.*

*Dr. G. Bunge, in Zeitschrift f. Physiologische Chemie. Bd.* 9.

UNIVERSITY OF MARYLAND, FACULTY OF PHYSIC, DEPART MENT OF DENTAL SURGERY.

BALTIMORE, July 28, 1885.

*To "The National Association of Dental Faculties."*

GENTLEMEN:—The Faculty of the University of Maryland, Dental Department, desire to acknowledge the receipt of the "Transactions of the National Association of Dental Faculties," and also the notice of the meeting to be held in Chicago July 31st, 1885, with Article VII. of the Constitution.

I am directed by the Faculty of the University of Maryland, Dental Department, to inform your Association that they will cheerfully comply with all the requirements relating to the graduation of students in dentistry, as adopted at the meeting held in New York City and Saratoga, in August, 1884, so far as refers to *two* full courses of five months each in separate years, etc., and have published the same in our Annual Catalogue of 1885, copies of which have been mailed to the President and Secretary of your Association. At the same time, however, the Faculty of the University of Maryland, Dental Department, deem it inexpedient to join the "National Association of Dental Faculties," for the reason that they believe the present curriculum of study, etc., in the University of Maryland, to be superior to any graded course of study, such as is obligatory upon all dental schools joining your Association, for the reason that it (the graded course) restricts the junior students to the study of dental mechanism alone for the one session of the two comprising the full course, to the exclusion of operative dentistry, and therefore affords dental students the advantage of but one session in the acquirement of a knowledge of a branch of our science for which the time of two sessions is not too long.

The Faculty of the University of Maryland, Dental Department, contend that students in dental schools should have all the advantages possible of the *two* sessions in *operative* as well as in mechanical dentistry, and that the adoption of such a graded course as that required by your Association, and which restricts the dental student in the manner referred to, must be *retrogressive* instead of progressive in dental education. Respectfully, etc.,

FERDINAND J. S. GORGAS,
Dean of Dental Department, University of Maryland.

### A PECULIAR CASE.

NOTE.—Close the eyes, listen attentively to the reading of the following, and see if you cannot easily imagine yourself in a Dental Society Meeting while Dr. So and So is propounding some of his lucid themes.

John Thompson was ill with malarial fever, and I prescribed for him Quinia Sulphas. The most peculiar circumstance about this case was as follows: John, as a matter of fact, had to remain in the house, and as he had nothing else to do, he held their ten-months-old baby on his lap a considerable portion of the time. During the four weeks that John was sick the baby had four teeth peep through the gums. The question at once arose in my mind, how did the quinine act to produce this result? After making a few circus rings around my bump of superlativeness, I evolved the following attenuated explanation. Naturally the child would inhale more or less of John's breath. John was taking the medicine, and of course he had a quinine-laden respiration. The child inhaled this, the quinine-germs came into contact with the gums and produced an evolution of the teeth. Or, to be more explicit, the teeth are composed of a substance that has sufficient density to produce a solution of continuity of a softer structure. The quinine-germs have a peculiar elective affinity for the teeth of children, they being rounded and located *subgumma*. The quinine-germs, being attracted by the magnetism that flows from the teeth to the food to assist in preparing it for the circulation of the liver, accumulate upon the gums over the tooth. In union there is strength, and as these germs increase in number at this place they form a powerful battery, and draw the tooth to a point, and the point pierces the solution of continuity in the gum. In the solution of this heretofore unexplained mystery we have made a wonderful discovery, and wish to call the attention of the faculty to this seeming *rara avis.—Cincinnati Lancet and Clinic.*

---

### A SPECIMEN.

There are some very illiterate dentists, but it is doubtful if the following letter from a physician could be paralleled. We print it *verbatim et spellatim*, but it is impossible to reproduce the wonderful chirography. Yet the man who is capable of producing such a letter as this is the confidential professional adviser of supposably rational human beings, and is entrusted with the care of human life:

C—— Mo

July 25 1885 Mr P—— Dr

Sir i See a Piec in the Pape about the Dentel asosatin Pleas tell me if a practising physician Can Be Come a member of your order i am a Doctor and Druggist yours as Ever Dr I N————

P o Box 66

### FERMENTS.

There are two great classes of ferments.

1st, The organized or formed ferments (equivalent to micro-organisms), sometimes called the true ferments.

2d, The unorganized, or unformed, or chemical ferments.

The first class comprises all the vegetable organisms that induce the fermentative process.

The second class includes:

| | |
|---|---|
| (*a.*)—Sugar-forming ferments ........ | Diastase.<br>Ptyaline.<br>Myrosine.<br>Emulsine.<br>Invertine, etc. |
| (*b.*)—Peptone-forming ferments....... | Pepsine.<br>Trypsine.<br>Papaine, etc. |
| (*c.*)—Albumen-forming ferments..... | Found in the glands of the Stomach. |
| (*d.*)—Glycerine-forming, or fat-splitting ferments. | Found in the secretions of the glands of the stomach, and certain intestinal glands. |
| (*e.*)—Ammonia-forming ferments.... | Active in the formation of Urea. |

In some of these fermentations acids may be formed, such as Hydrocyanic, by the action of emulsine on amygdaline; fatty acids, by the action of glycerine-forming ferments on neutral fats; salicylic acid, by the action of emulsine on the glycocide of salicylic acid, etc., etc., etc. As I showed in the early part of 1884, it is not probable that any fermentation which produces acid, other than that inaugurated by micro-organisms, occurs in the mouth.—*Extract from a letter of Dr. W. D. Miller.*

---

### PERSONAL.

A letter from our colleague, Dr. Bodecker, dated Aug. 4th, and mailed at Kreutsnach, Germany, conveys the information that his health is materally improved. He will spend some time at St. Moritz, Switzerland, which he hopes will complete his restoration. He will return about October 1st, and Dr. W. Herbst, of Bremen, will probably accompany him on a visit to this country.

### THE LAW OF FINDING.

The law of finding, says a writer, is this:—The finder has a clear title against the world, except the owner. The proprietor of a coach, or a railroad car, or a ship, has no right to demand the property on a premise. Such proprietors may make regulations in regard to lost property which will bind their employees, but they cannot bind the public. The law of finding was declared by the King's bench 100 years ago, in a case in which the facts were these: A person found a wallet containing a sum of money on a shop floor. He handed the wallet and contents to the shopkeeper, to be returned to the owner. After three years, during which the owner did not call for his property, the finder demanded the wallet and the money from the shopkeeper. The latter refused to deliver them, upon the ground that they were found on his premises. The former then sued the shopkeeper, and it was held, as above set forth, that, against all the world but the owner, the title of the finder is perfect, and the finder has been held to stand in the place of the owner, so that he was permitted to prevail in an action against a person who found an article which the plaintiff had originally found, but subsequently lost. The police have no special rights in regard to articles lost, unle s those rights are conferred by statute. Receivers of articles stolen are trustees for the owner or finder. They have no power in the absence of special statute to keep an article against the finder, any more than a finder has to retain an article against the owner.

---

### ANÆSTHETICS—GOOD, BAD AND INDIFFERENT.

Dr. B. W. Richardson has recently published a summary of the anæsthetics, from which we condense the following:—

Considered safe: ethyl bromide, ethyl chloride, ether, ethene (olefiant gas), ethene chloride, methyl bromide, methyl chloride, methyl ether, methene chloride, methane (marsh gas), nitrous oxide.

Of doubtful value: amylene, amyl chloride, butyl chloride, benzene (benzol), carbon disulphide, carbon dioxide, carbon tetrachloride, methyl alcohol, methylal, spirits of turpentine.

Dangerous: amyl hydride, butyl hydride, carbon monoxide, ethyl hydride. Chloroform and ethene dichloride are regarded as useful, but requiring care. Dr. Richardson considers methylic ether to be the safest of all the anæsthetics.

### DENTAL HYGIENE.

M. Gallippe, in a discussion before the *Societe de Medecine de Publique de Paris*, on dental hygiene in schools, insisted on the fact that overtaxing the brain by over-study in school affected dental growth. Among those students who work hard, the teeth become deteriorated a few weeks after their entry; caries is frequent among the successful pupils; the second dentition is frequently premature, and the teeth that appear are diseased. Among students who are really overworked, it frequently happens that the teeth begin to decay when hard work reaches its maximum at the time of examination, or those that are faulty grow worse, become very painful and have to be extracted.—*London Med. Record.*

### A DENTAL NOVELIST.

The *Dental Record* of London is publishing as a *feuilleton* a work of dental fiction, entitled "Thurley Tighe, the Life of a Student," by the author of "Vernon Galbray," a book, the publication of which made some stir in professional circles not many years since. So far "Thurley Tighe" proves to be of great interest. It is published anonymously, but we think we are betraying no confidence when we say to our American readers that the author is the venerated "Phosphor" of the English journals, Felix Weiss, L. D. S., the librarian of the Odontological Society of Great Britain. We publish a brief communication from him in this number.

### A NEW MECCA ON THE MAINE COAST.

The Underwood Spring at Falmouth Foreside is the scene of a remarkable pilgrimage. A wide-spread belief in the efficacy of its waters has taken possession of the inhabitants of the surrounding country, and a continuous procession of people, on foot and in wagons, with even carriages from the neighboring Portland, carry off the water, every conceivable vessel from a barrel to a milk-can being pressed into service for the purpose.—*Boston Post.*

### HONORS HONESTLY WON.

Michigan University has properly recognized a worthy son who has reflected honor upon his Alma Mater, by conferring the honorary degree of Doctor of Philosophy upon Prof. W. D. Miller, of Berlin. The German *Kultus Ministerium* has also made of him an *Approbirter Zahnarzt.* These are honors conferred upon an American dentist at which every American dentist should rejoice.

## Figures and Tables

**FIG. 1. f1:**
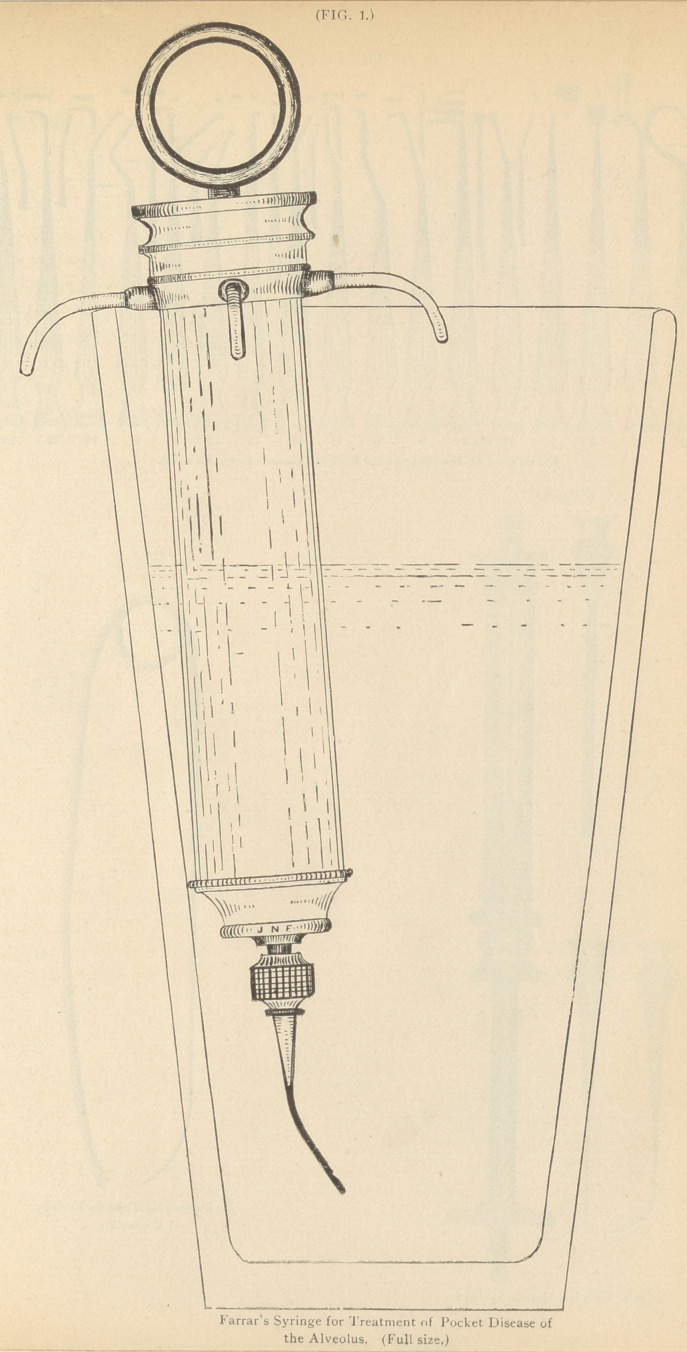


**FIG. 2. f2:**
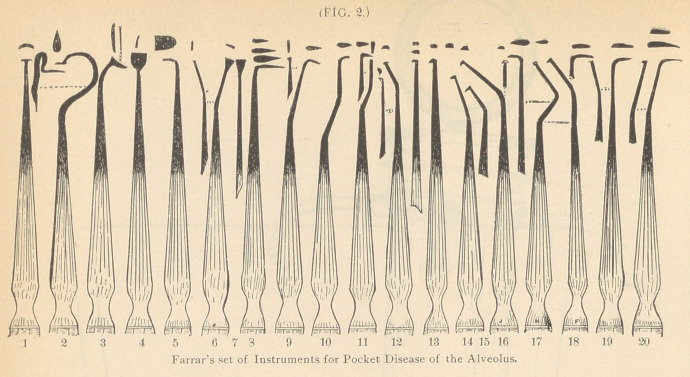


**FIG. 3. f3:**
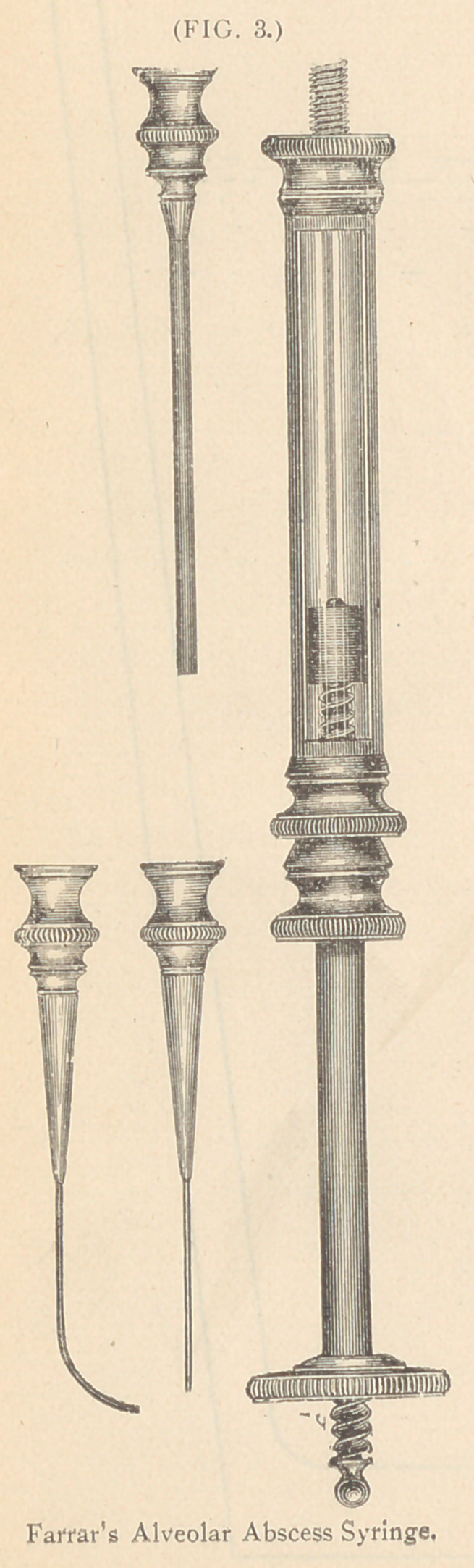


**FIG. 4. f4:**